# Perioperative chemotherapy for clinical stage IIIB gallbladder cancer: a review of the National Cancer Database

**DOI:** 10.1007/s10147-026-03051-w

**Published:** 2026-05-28

**Authors:** Jennifer Wylie, Grey Leonard, Brian White, Clancy Clark, Edward Levine, Perry Shen

**Affiliations:** 1 Wake Forest School of Medicine, Winston Salem, NC, USA; 2 Premier Surgical Associates at Fort Sanders Regional Medical Center, Knoxville, TN, USA; 3 Virginia Mason Medical Center, Seattle, WA, USA; 4 Medical Center Blvd Winston Salem, Winston-Salem 27157, NC, USA

**Keywords:** Gallbladder cancer, National Cancer Database, chemotherapy, surgery

## Abstract

**Background:**

Gallbladder cancer is rare with high mortality and limited treatments. Patients with IIIB disease (T1–3, N1, M0) are typically not offered resection and may receive palliative chemotherapy. This study evaluates survival of IIIB patients undergoing surgery with or without systemic therapy.

**Methods:**

Patients with stage IIIB gallbladder cancer who underwent curative-intent surgery were identified from the National Cancer Database (2004–2020). Outcomes were analyzed by chemotherapy sequence using Kaplan-Meier curves and Cox proportional hazards models. Lymphadenectomy type and margin status were also compared.

**Results:**

409 patients were included. Mean age was 60 in neoadjuvant, 65 in adjuvant and 72 in no chemotherapy classes (*p* < 0.01). The median overall survival (OS) in months was 33.8 (95% CI 23.8 - NA) in neoadjuvant, 22.6 (95% CI 19.8–27.2) in adjuvant and 9.4 (95% CI 7.3–14.3) in no chemotherapy groups (*p* < 0.01). Compared to adjuvant therapy, hazard ratios (HR) for OS were 0.76 (95% CI 0.46–1.26, *p* = 0.29) for neoadjuvant and 1.87 (95% CI 1.45–2.42, *p* < 0.01) for no chemotherapy. Age, tumor size, number of positive regional nodes, and positive margins (R1) were significant covariables. Regional lymphadenectomies were performed in 79%, 87%, and 84% for neoadjuvant, adjuvant, and no chemotherapy classes. R0 margins were obtained in 86%, 76%, and 81%, respectively.

**Conclusion:**

Surgery with systemic therapy should be considered in stage IIIB gallbladder cancer. Perioperative chemotherapy was associated with longer survival but occurred more in younger patients. Perioperative chemotherapy, particularly neoadjuvant, may benefit appropriately selected patients.

## Introduction

Gallbladder carcinoma is a rare but serious diagnosis with an estimated incidence of 1.31 per 100,000 persons in the United States with female predominance [1]. When grouped, as in many studies, with biliary tract cancer, approximately 20% of patients are surgical candidates and 5-year overall survival (OS) is less than 10% [2]. Survival in gallbladder cancer patients who undergo surgery varies greatly by stage [3], while 40% are diagnosed with distant disease on presentation [1]. For resectable disease, surgery is considered the standard of care [4, 5]. For incidental cancer, stage T1a, discovered after cholecystectomy, no further surgery is recommended whereas patients with resectable advanced stage disease benefit from re-resection, typically with en bloc hepatectomy and portal lymphadenectomy [6]. Locally advanced disease, discovered on imaging prior to surgery represents an uncommon presentation of a rare disease and there is a paucity of data to guide decision making and guidelines are correspondingly vague. For these cases deemed resectable, the National Comprehensive Cancer Network (NCCN) guidelines [7] state “For locoregionally advanced disease, consider neoadjuvant systemic therapy to rule out rapid progression and avoid futile surgery. There are limited clinical trial data to define a standard regimen or definitive benefit”. The purpose of this study is to examine real-world data on the current treatment strategies and associated outcomes for surgical patients diagnosed with clinical stage IIIB (T1–3, N1, M0) gallbladder cancer, aiming to address this critical knowledge gap and inform future decision making.

## Patients and methods

This study was approved by the Wake Forest University School of Medicine IRB. The gallbladder carcinoma NCDB was analyzed, including data from 2004 to 2020. The initial cohort of patients with available survival data included 45,397 patients. Patients were chosen who had clinical stage IIIB disease on the assumption that clinical included patients who had not previously undergone a cancer operation with curative intent. Clinical lymph node positivity in the NCDB is defined by AJCC cN staging and is based on pre-treatment clinical assessment including physical examination, imaging, and biopsy information when available. Next, only patients who underwent primary site surgery were included. This cohort included sequence data for chemotherapy, coalesced into three groups: neoadjuvant, adjuvant, and no chemotherapy. A fourth category, chemotherapy before and after, was included in the neoadjuvant group. Pertinent clinical data was included per the authors’ experiences and missing data was imputed using single predictive mean matching imputation from the MICE package in R [8]. Kaplan-Meier plots were created using survival data in months. A multivariate Cox proportional hazards model was created using selected covariates. R statistical software was used for all analysis. As a sensitivity analysis, inverse probability weighting (IPW) was performed to adjust for potential confounding across chemotherapy classes. Stabilized weights were estimated using a covariate balancing propensity score (CBPS) model with age, sex, Charlson-Deyo score, and year of diagnosis as covariates; the IPW-weighted Cox model employed robust sandwich variance estimators.

## Results

**Fig. 1:**
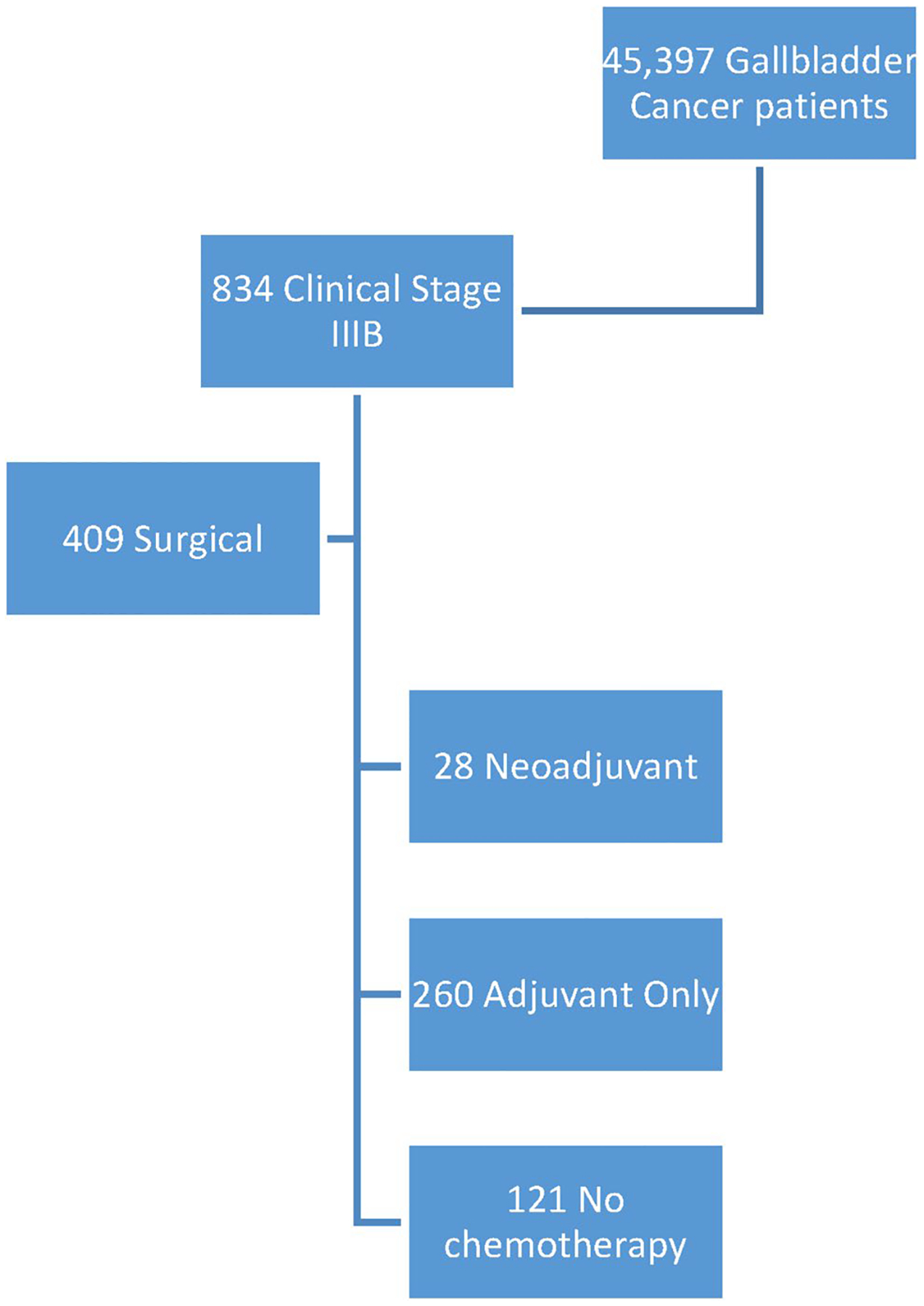
CONSORT diagram illustrating patient selection and treatment allocation. Of 45,397 patients diagnosed with gallbladder cancer, 834 were identified with clinical stage IIIB disease. Among these, 409 underwent surgical intervention. Chemotherapy distribution included 28 patients receiving neoadjuvant therapy, 260 receiving adjuvant therapy only, and 121 receiving no chemotherapy

Of the available data, 834 (1.8%) patients with clinical T1–3, N1 and M0, corresponding to stage IIIB disease, were included. 409 (49%) of these patients underwent surgery, though the specifics of the extent of surgery, excluding regional lymphadenectomy, were not available. Using sequence data, there were 28 patients in the neoadjuvant group, 260 in the adjuvant only group and 121 in the no chemotherapy group (Fig. 1).

**Fig. 2:**
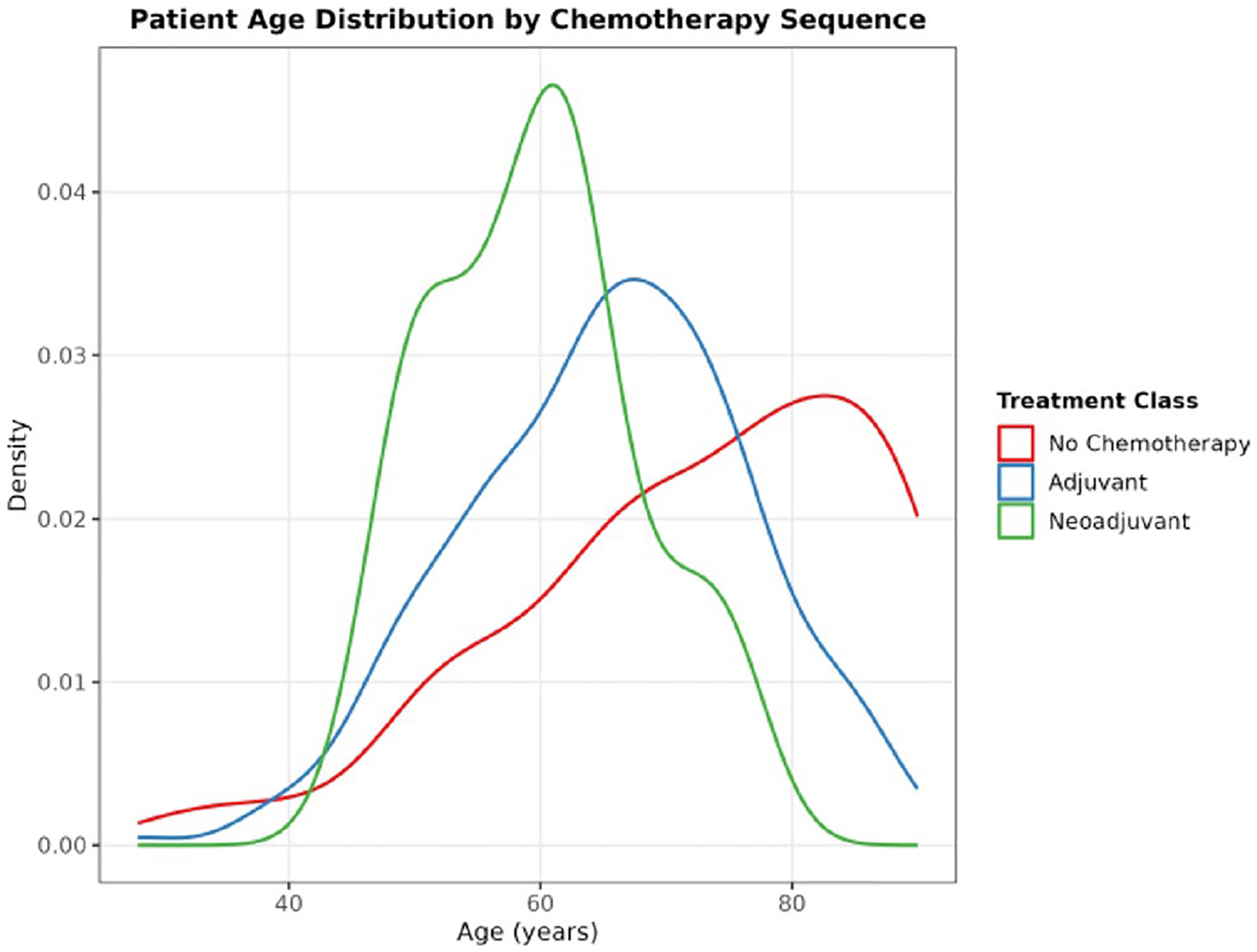
Patient age distribution by chemotherapy sequence class. Kernel density plots illustrate age patterns for patients receiving no chemotherapy (red), adjuvant chemotherapy (blue), and neoadjuvant chemotherapy (green)

**Fig. 3:**
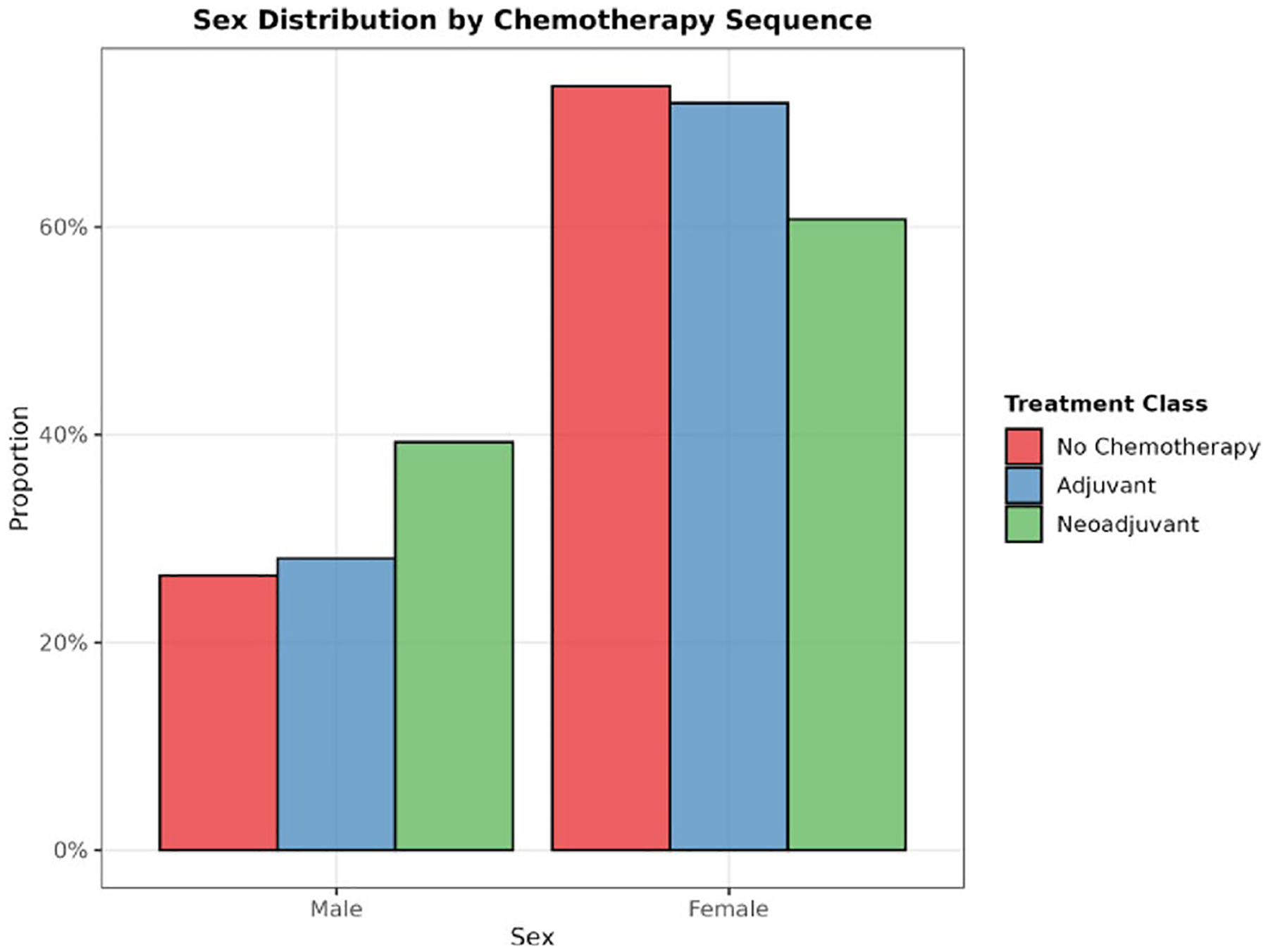
Patient sex distribution by chemotherapy sequence class. Bar plots represent the proportion of male and female patients within each treatment group: no chemotherapy (red), adjuvant chemotherapy (blue), and neoadjuvant chemotherapy (green)

There was a significant age difference among the sequence data. Mean ages for neoadjuvant, adjuvant and no chemotherapy were 60, 65 and 72, respectively. Figure 2 demonstrates a density plot of the ages by class. There was a sex discrepancy with female predominance overall (71.6%) with similar proportions by the sequence classes (Fig. 3). All three groups had similar Charlson-Deyo scores. Rates of regional lymphadenectomy were similar across the groups, 79% for neoadjuvant, 87% for adjuvant and 84% for the no chemotherapy groups. The overall rate of lymphadenectomy was 85.8%. There were fewer regional lymph nodes harvested in the no chemotherapy group (3.2, *p* < 0.01) than the neoadjuvant and adjuvant groups (4.3 and 4.0). Number of positive regional nodes and rates of R0 resection did not differ by sequence class. Comparison between groups are summarized in Table 1.

**Fig. 4:**
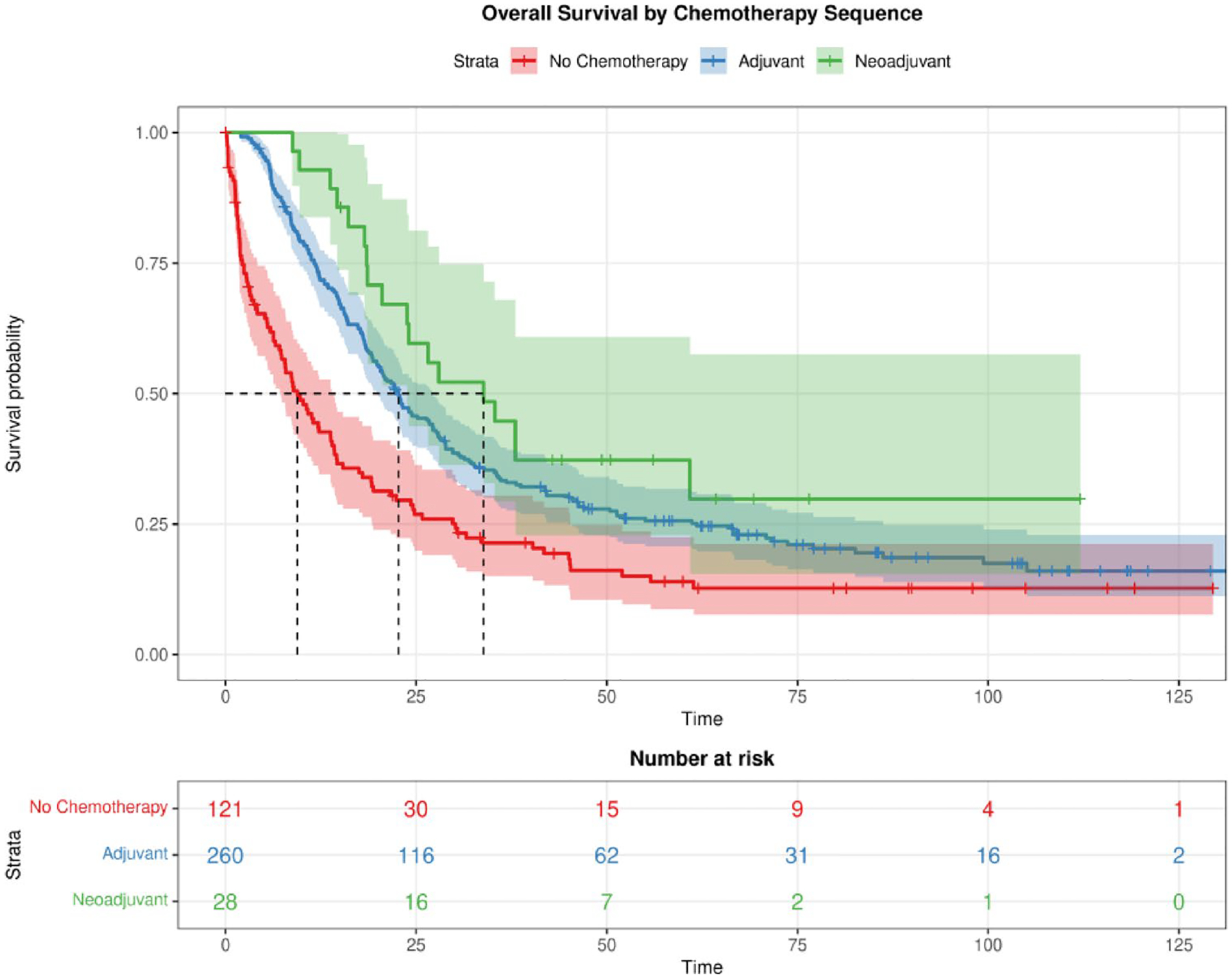
Kaplan-Meier survival estimates in months stratified by chemotherapy sequence class: no chemotherapy (red), adjuvant (blue), and neoadjuvant (green). Shaded areas represent 95% confidence intervals. Dotted lines indicate median survival time in months for each group. Numbers at risk are shown below the x-axis

On univariate analysis, there were differences in median OS in months across the sequence classes. Median OS was 33.8 (95% CI 23.8 - NA), 22.7 (95% CI 19.9–27.2) and 9.4 (95% CI 7.3–14.3), *p* < 0.01, for neoadjuvant, adjuvant and no chemotherapy groups respectively. Kaplan-Meier plots are shown in Fig. 4. On univariate Cox modeling with adjuvant as the reference, the neoadjuvant group had a HR of 0.71 (95% CI 0.44–1.15, *p* = 0.165). When the no chemotherapy group was the reference, both neoadjuvant and adjuvant groups were associated with less hazard, HR 0.41 (95% CI 0.25–0.67, *p* < 0.01) and HR 0.57 (95% CI 0.45–0.73, *p* < 0.01).

**Fig. 5:**
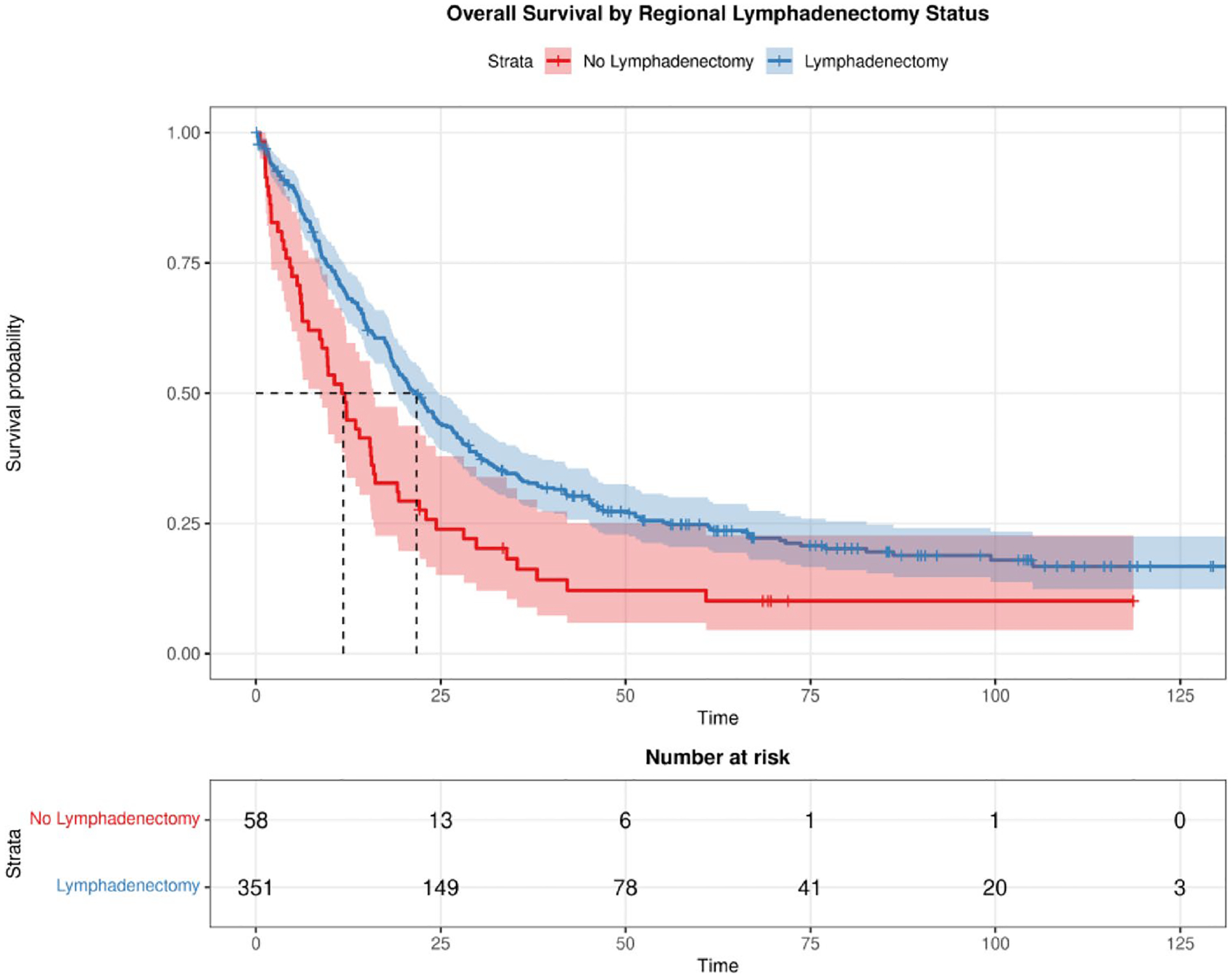
Kaplan-Meier survival estimates in months stratified by regional lymphadenectomy status. Patients without lymphadenectomy are shown in red and those with lymphadenectomy in blue. Shaded areas represent 95% confidence intervals. Dotted lines indicate median survival time in months for each group. Numbers at risk are shown below the x-axis

Again, on univariate analysis, there were differences in median OS in months between patients that had a regional lymphadenectomy and those that did not. Median OS was 21.7 (95% CI 18.8–24.6) with lymphadenectomy and 11.8 (95% CI 8.6–15.9) without (*p* < 0.01). On Cox modeling this corresponded to a HR of 0.58 (95% CI 0.43–0.78, *p* < 0.01). A Kaplan-Meier plot is shown in Fig. 5.

A multivariate analysis was performed with Cox modeling including the prespecified covariates and the results are summarized in Table 2. Significant covariates for survival included patient class, age, tumor size (mm), number of positive regional nodes and margins. With adjuvant only as the reference, the HR for neoadjuvant chemotherapy was 0.76 (95% CI 0.46–1.26, *p* = 0.29) and 1.87 (95% CI 1.45–2.42, *p* < 0.01) for no chemotherapy. With no chemotherapy as the reference the HR for neoadjuvant and adjuvant groups were 0.41 (95% CI 0.24–0.69, *p* < 0.01) and 0.53 (95% CI 0.41–0.69, *p* < 0.01). Regional lymphadenectomy approached statistical significance with a HR of 0.75 (95% CI 0.53–1.05, *p* = 0.10).

**Fig. 6:**
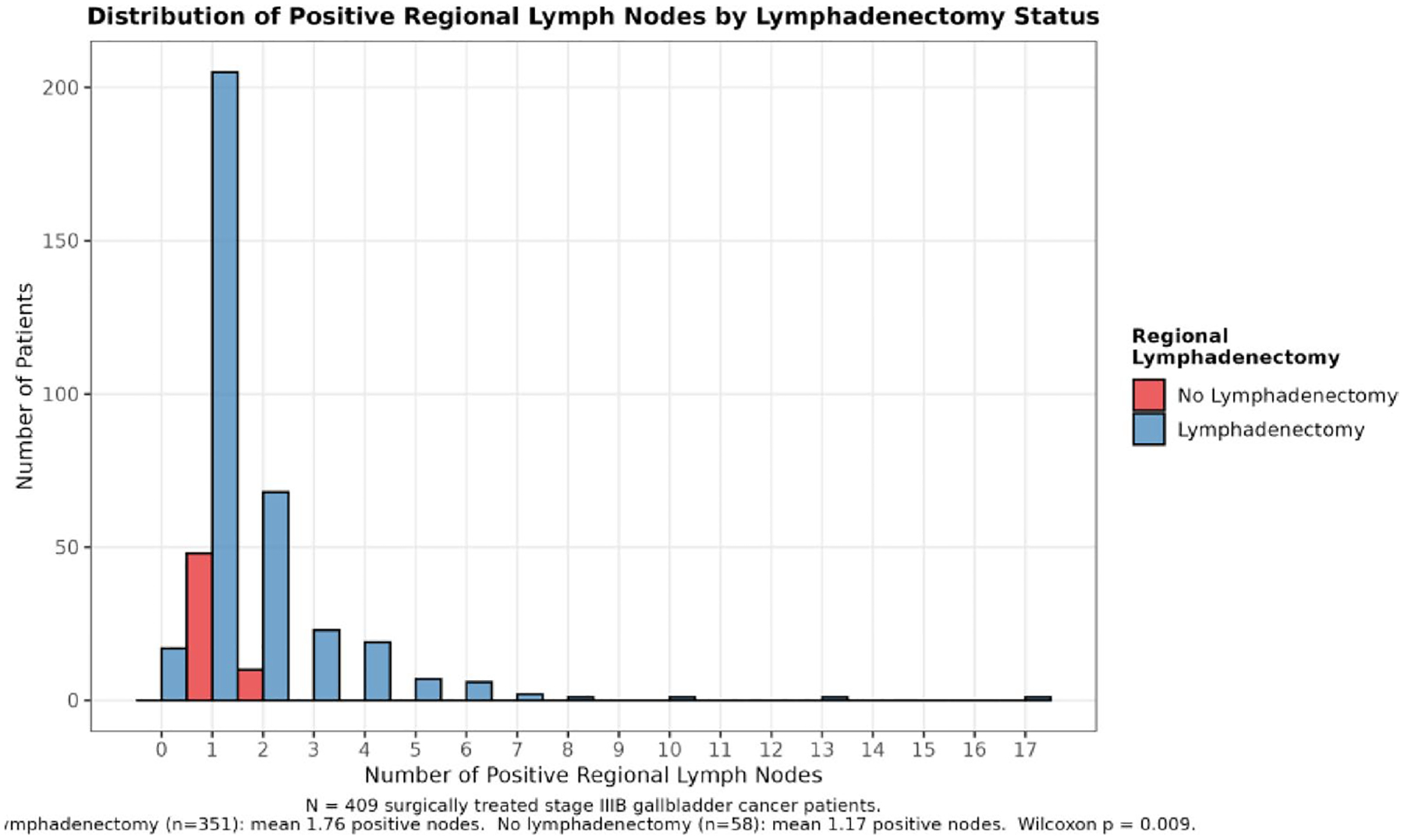
Distribution of the number of positive regional lymph nodes stratified by lymphadenectomy status. Among 409 patients with clinical stage IIIB gallbladder cancer who underwent surgery. Lymphadenectomy group (*n* = 351): mean 1.76 positive nodes. No lymphadenectomy group (*n* = 58): mean 1.17 positive nodes (Wilcoxon *p* = 0.009)

Of the cohort of surgical, clinical stage IIIB patients, 17 (4.2%) were found to have no pathologically positive lymph nodes. The regional lymphadenectomy group averaged 1.76 positive nodes compared to 1.21 in those that did not have regional lymphadenectomy. The distribution of positive nodes by lymphadenectomy status is shown in Fig. 6.

The number of positive lymph nodes differed significantly between the lymphadenectomy group (mean 1.76, SD 1.70) and the non-lymphadenectomy group (mean 1.21; Wilcoxon *p* = 0.009). Across chemotherapy classes, the number of positive lymph nodes did not differ significantly (Kruskal-Wallis *p* = 0.08). However, the rate of pathological node positivity varied by class (Fisher exact *p* = 0.004), with 82.1% of neoadjuvant patients having positive nodes compared to 96.2% in the adjuvant and 98.3% in the no chemotherapy groups. Margin status did not differ significantly across groups (Fisher exact *p* = 0.71).

**Fig. 7:**
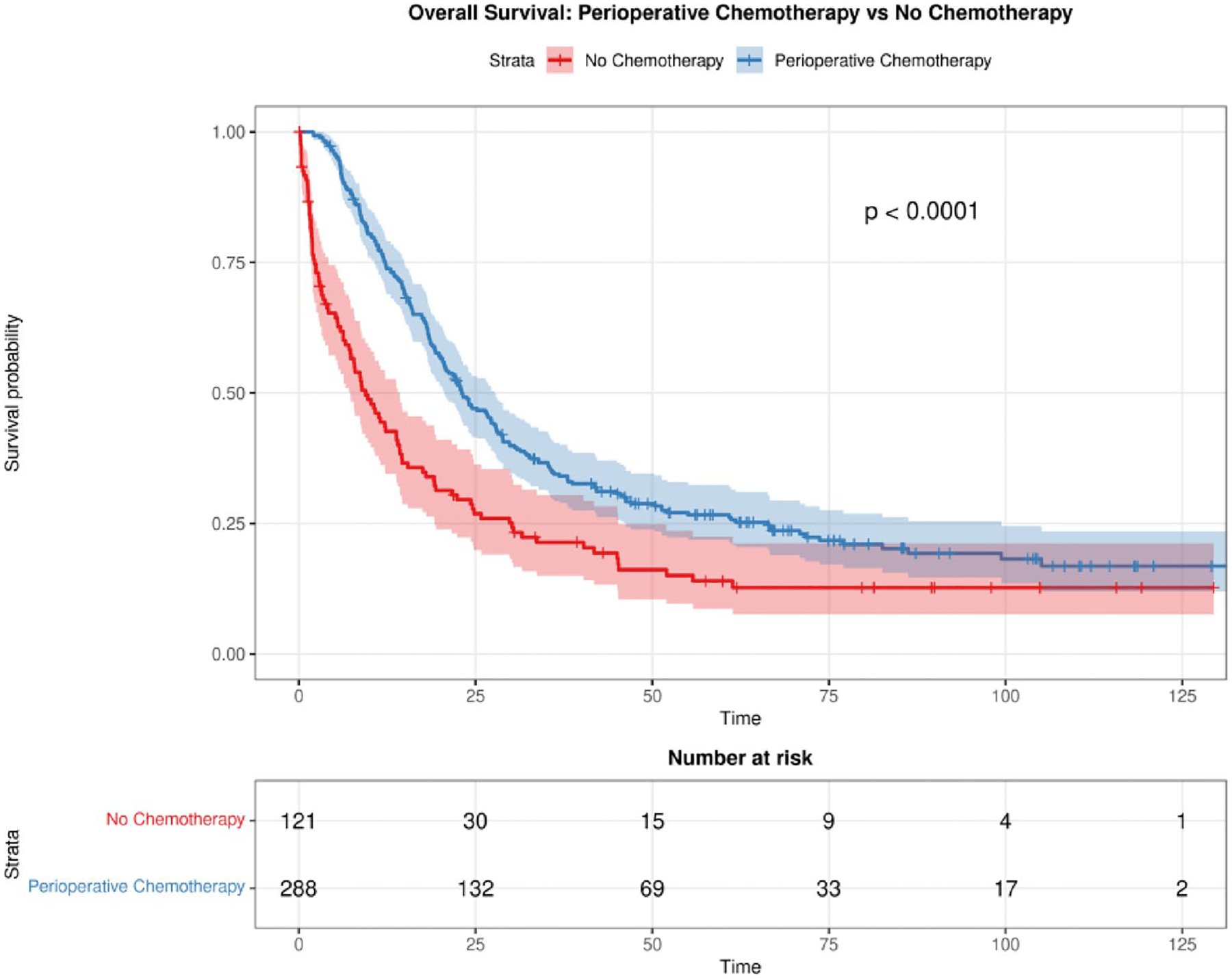
Kaplan-Meier survival estimates comparing combined perioperative chemotherapy (*n* = 288) versus no chemotherapy (*n* = 121). Shaded areas represent 95% confidence intervals. Numbers at risk are shown below the x-axis. Log-rank *p* < 0.001

When neoadjuvant and adjuvant groups were combined, perioperative chemotherapy (*n* = 288) was associated with significantly longer overall survival compared to no chemotherapy (*n* = 121; median OS 23.0 vs. 9.4 months, log-rank *p* < 0.001; Fig. 7). On multivariate analysis, perioperative chemotherapy was associated with a HR of 0.48 (95% CI 0.37–0.62, *p* < 0.001).

**Fig. 8:**
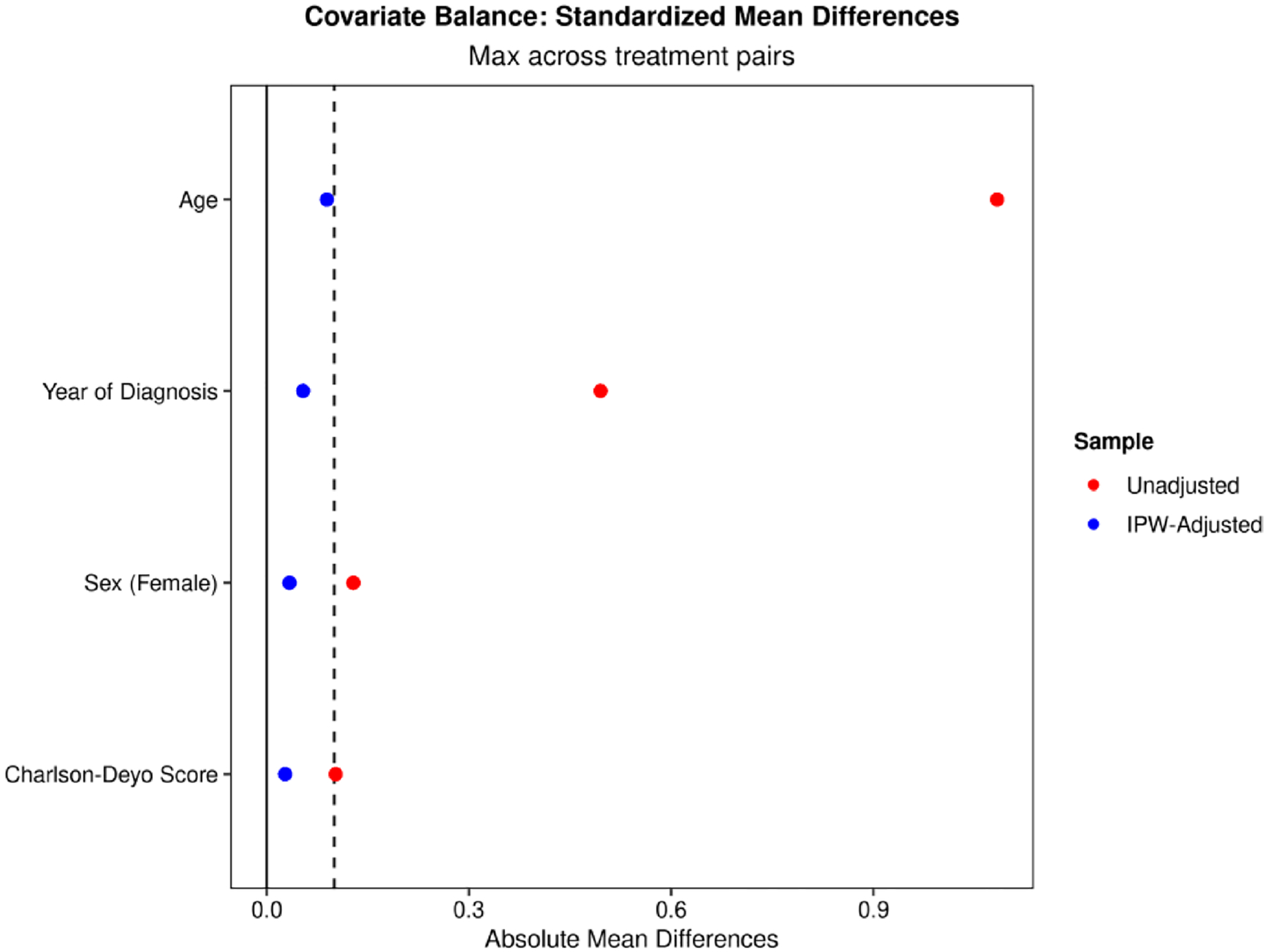
Covariate balance plot showing absolute standardized mean differences before (red) and after (blue) inverse probability weighting. The dashed line indicates the balance threshold of 0.10

**Fig. 9:**
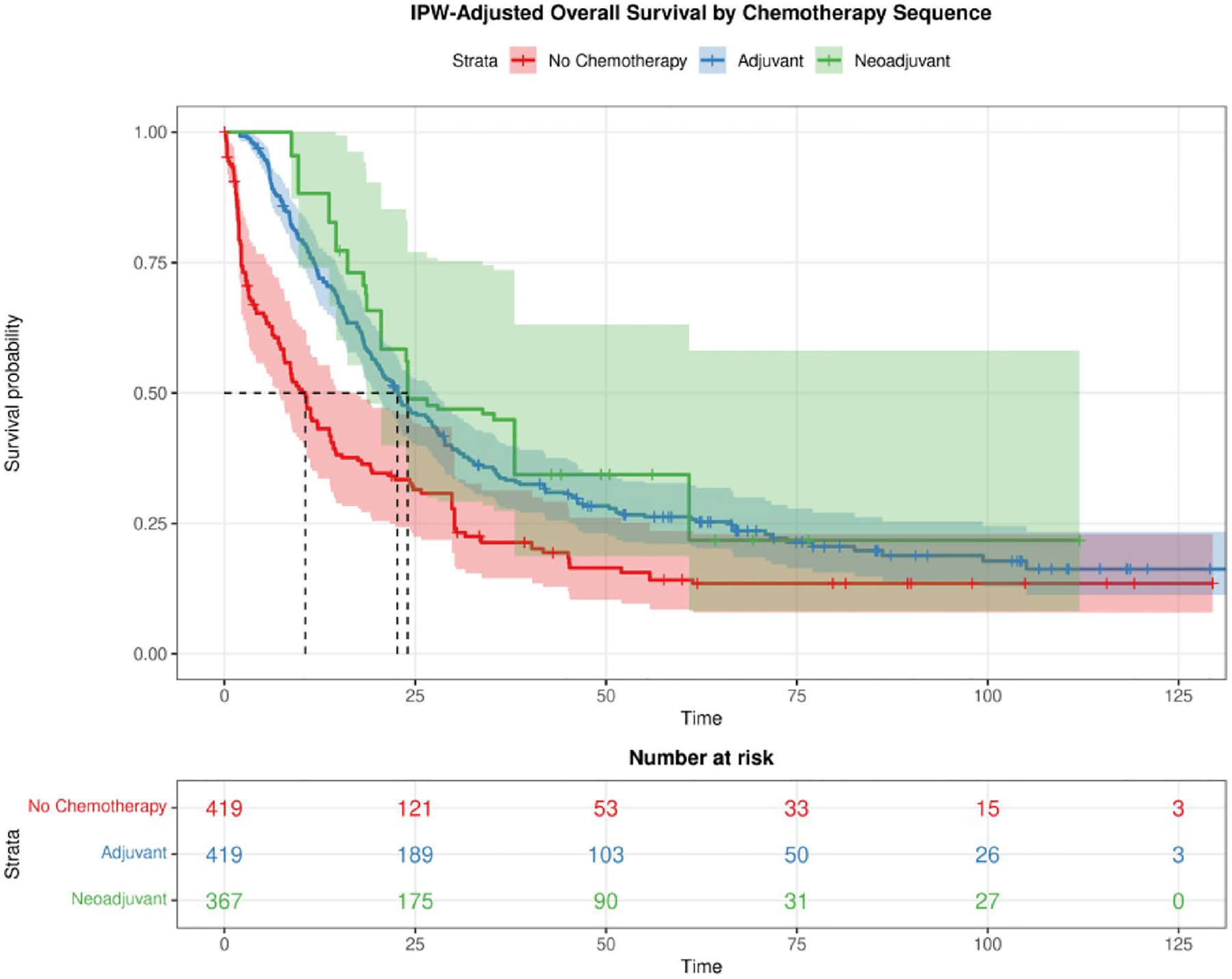
IPW-adjusted Kaplan-Meier survival estimates stratified by chemotherapy sequence class. Numbers at risk are shown below the x-axis

On IPW-weighted sensitivity analysis, adequate covariate balance was achieved across all treatment pairs, with all standardized mean differences below 0.10 (Fig. 8). The IPW-weighted Kaplan-Meier survival curve (Fig. 9) and multivariate Cox model yielded results consistent with the primary analysis. With no chemotherapy as reference, HRs were 0.44 (95% CI 0.33–0.58, *p* < 0.001) for adjuvant and 0.38 (95% CI 0.23–0.62, *p* < 0.001) for neoadjuvant chemotherapy (Table 3), confirming that the survival advantage associated with perioperative chemotherapy is robust to adjustment for baseline confounding.

## Discussion

Gallbladder carcinoma remains a bleak diagnosis, though advances in chemotherapy continue to show some promise for biliary tract cancer [2, 9]. While rare, surgical standards have been developed to improve quality and optimize outcomes. In 2022, the American College of Surgeons published operative standards for hepatobiliary cancer [10]. The gallbladder carcinoma recommendations included selective staging laparoscopy, R0 resections, routine portal (regional) lymphadenectomy (with a suggestion of 3–6 nodes) and allowed departure from routine port site excisions for incidentally discovered cancer. As mentioned previously, the ancillary treatment for stage IIIB carcinoma remains poorly defined, resulting in heterogenous approaches to chemotherapy, as seen in this study. Given the specificity of clinically stage IIIB gallbladder carcinoma, clinical trial data is lacking and often require extrapolation. Some recent and ongoing trials have attempted to delineate the role of neoadjuvant chemotherapy in gallbladder cancer [11], but these vary in both gallbladder specificity [12] and the definition of locally advanced [13]. Other trials, including NCT04480190 [14] were designed to examine neoadjuvant chemoradiation in resectable biliary adenocarcinoma. The NCT04559139 trial is designed to examine role of perioperative chemotherapy for gallbladder cancer in stage II to IIIB, but is not scheduled to conclude until 2029 [15]. Consequently, there is no clear consensus on the optimal chemotherapy sequencing or specific regimen. In this study, the NCDB was used in a retrospective analysis to provide real-world insight into the treatment patterns and outcomes in this rare patient population, addressing this critical gap.

Since the BILCAP trial in 2019 [2], there has been more emphasis on adjuvant treatment for biliary tract cancer, including gallbladder carcinoma. This is reflected in the current NCCN guidelines (version 3.2024) and was adopted as part of the ASCO Clinical Practice Guideline in April 2019. Yet the benefit of adjuvant chemotherapy is far from certain. In the subgroup analysis from BILCAP for muscle invasive gallbladder carcinoma, the impact for capecitabine was modest with a HR for OS of 0.84 (95% CI 0.43–1.63) compared to observation alone. Prior to this, the PRODIGE 12/ACCORD 18 study, examining gemcitabine and oxaliplatin following resection for biliary tract cancers, failed to show a benefit in relapse free survival [16]. However, the JCOG1202: ASCOT trial [17], presented in 2022, evaluated the benefit of S-1, an oral fluoropyrimidine derivative, as adjuvant therapy for resected biliary tract cancer. They reported a HR of 0.69 for OS for all patients in the treatment group (95% CI 0.51–0.94, *p* = 0.008). For further data as to the question of adjuvant therapy in gallbladder carcinoma, we await the results of the ACTICCA-1 trial [18], scheduled to conclude in 2024. However, given the heterogeneity of published results, there remains ambiguity about the benefit of adjuvant therapy for gallbladder cancer.

There are fewer data to guide decisions about neoadjuvant therapy for gallbladder carcinoma. The JCOG1920:NABICAT trial [19], currently enrolling, is seeking to assess the value of neoadjuvant gemcitabine, cisplatin and S-1 vs. surgery first for resectable biliary tract cancer. For gallbladder cancer this includes those with stage IIIA-IVA disease. This regimen was based on the KHBO1401-MITSUBA [12] data which demonstrated a survival benefit in advanced, unresectable biliary tract cancer of gemcitabine, cisplatin and S-1 over gemcitabine and cisplatin alone. Additionally, the AIO/CALGP/ACO-GAIN trial [20], also ongoing, is examining the role of neoadjuvant/perioperative chemotherapy in biliary tract cancer with gemcitabine and cisplatin.

The NCBD data provides a picture of past treatment and quality strategies for patients presenting with clinical stage IIIB disease. We could detect 409 patients matching this description with variance in chemotherapy strategies and lymphadenectomy rates. Of the patients with clinical stage IIIB disease, only 49% received surgery. It is undeterminable from the NCDB data why the other 51% did not receive surgery. Perhaps the surgeons thought surgery was futile at this stage, or they considered the patient unresectable. However, of those who received surgery, 86% received a regional lymphadenectomy. We found a trend of association of regional lymphadenectomy with survival, though losing statistical significance on the multivariate model (HR 0.75, *p* = 0.10). However, following IPW-adjusted analysis, this association strengthened (HR 0.61, *p* = 0.02), indicating that baseline imbalance may have attenuated the unweighted estimate. Furthermore, this trend persisted despite a similar number of positive lymph nodes across chemotherapy classes and fewer nodes examined in the no-chemotherapy group, thus suggesting the prognostic relevance of lymphadenectomy extends beyond absolute nodal disease burden. One possible explanation is that more extensive nodal evaluation may facilitate enhanced local regional control, improve staging accuracy, and limit under-staging. It is important to note that adequate lymphadenectomies are often performed by specialists at high-volume centers where improved outcomes may reflect a variety of factors associated with institutional experience. However, surgeon and center-level volume metrics were not available in the NCBD for adjustment in our analysis. Additionally, our results demonstrated that the number of positive nodes found was also independently associated with increased risk of death. Thus, despite limitations in the scope of our data, these findings support that both extent of nodal evaluation and nodal burden play a role in patient outcomes, highlighting the importance of adherence to the American College of Surgeons (ACS) standards for complete regional lymphadenectomy to ensure high-quality care.

The initial statistical tests sought to determine a difference in hazard between the chemotherapy sequences of the neoadjuvant group and the adjuvant group. While there was some difference, this did not pass statistical significance. However, this may have been due to low numbers in the neoadjuvant group (n=28) compared to the adjuvant (n=260). What is clear is that some chemotherapy, either pre- or postoperative, was associated with less risk of death. This was made clear by making the no chemotherapy group the reference in statistical tests. This finding must be seen in the context of the increased age of the no chemotherapy group, as older adults are more likely to die at baseline and may not have been good candidates for chemotherapy. Age was also an independent risk factor for death on the multivariate model.

Additional hazards included the tumor size and margin positivity. This demonstrated the importance of surgical technique and the danger of advanced disease. These factors highlighted a theoretical reason for neoadjuvant therapy in attempting to shrink the tumor and/or arrest microscopic tumor spread. While the size of the tumors differed in the neoadjuvant group (39 mm) vs. the adjuvant (47 mm), this was not statistically significant. Likewise, the R0 rate in the neoadjuvant group was higher (86% vs. 76%), though not achieving statistical significance. We did however see a significant difference in pathological node positivity rate of 82.1% in the neoadjuvant group compared to 96.2% in the adjuvant group (Fisher *p* = 0.004). Thus, when considering the improved survival trend in patients receiving neoadjuvant therapy (HR 0.76, *p* = 0.29 with adjuvant as baseline and 0.41, *p* < 0.01 with no chemotherapy as baseline), it is possible that neoadjuvant therapy may lead to improved micro metastasis control or pathological downstaging. Given that these trends persist following adjustment for age, sex, Charlson-Deyo score, and year of diagnosis (HR 0.38, *p* < 0.001), patient selection or other possible confounders are less likely explanations; however, they remain difficult to rule out due to the lack of specificity of the NCBD dataset.

**Fig. 10:**
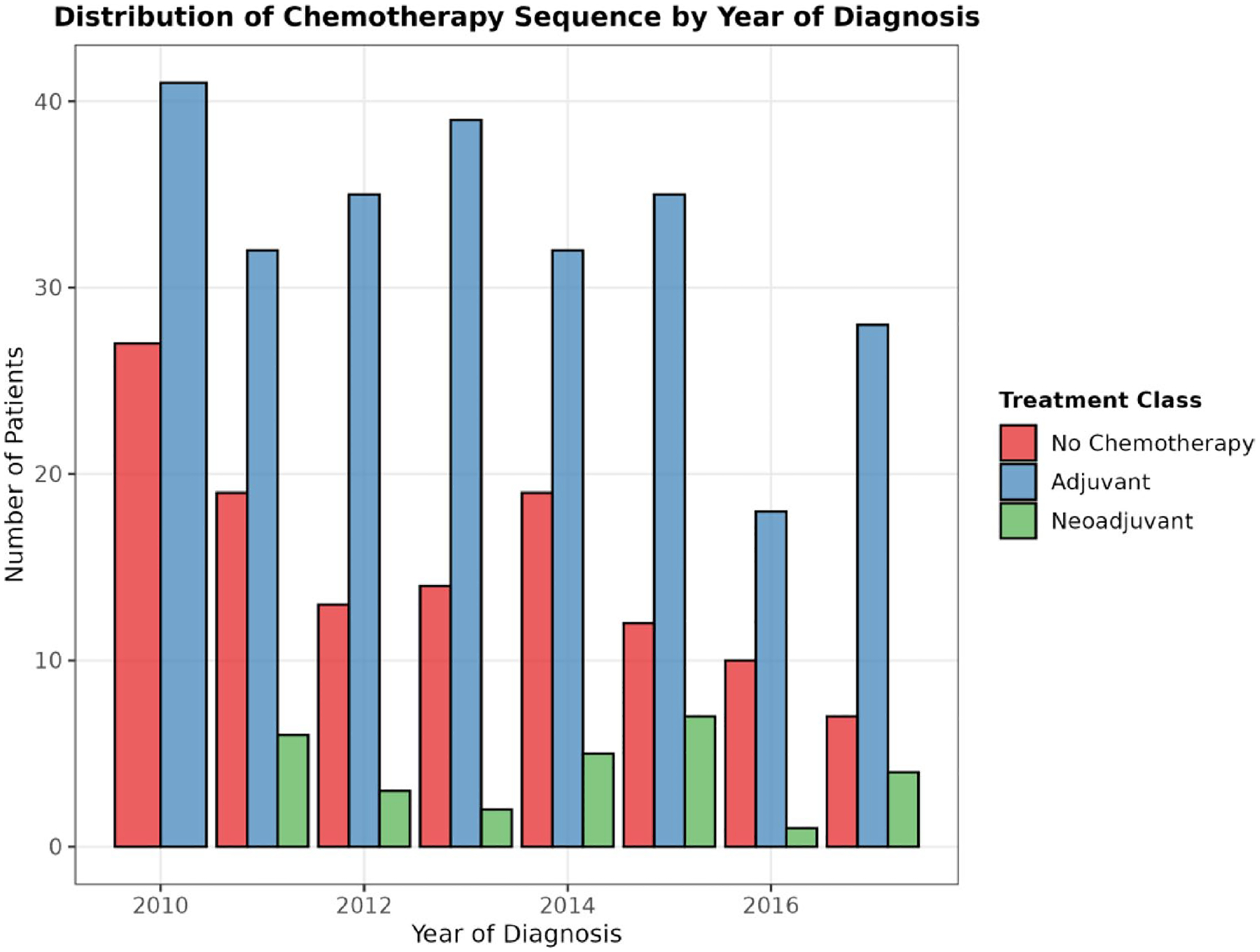
Distribution of chemotherapy sequence class by year of diagnosis (2010–2017)

This study was subject to several limitations. First, while the NCDB provided a wealth of data which was particularly helpful in studying a rare presentation of a rare disease, the data lacked detail and is subject to bias. For example, given the lack of pathological staging information, clinical staging was used which is prone to misclassification. Additionally, it was impossible to ascertain what cancer operation was performed, i.e. simple cholecystectomy vs. cholecystectomy with a IVb/V partial hepatectomy. The type or combination of chemotherapy was also absent, and the database contained different categories for perioperative chemotherapy, disparate from neoadjuvant or adjuvant only. As such, the neoadjuvant group included any patients who received neoadjuvant therapy, even if they also received adjuvant therapy. Second, despite the ample number of subjects within the NCDB, there were only 409 patients available that met selection criteria, placing limits on the statistical analysis. Third, the retrospective nature of the data invited room for potential selection bias and potential confounders, most notably the elevated age of the no chemotherapy group. Patients receiving chemotherapy were younger and likely had better performance status, which may explain survival differences. To address this concern, inverse probability weighting was performed as a sensitivity analysis, which demonstrated that the observed survival differences persisted after adjustment for age, sex, comorbidity, and year of diagnosis. Fourth, the range of the data collection spanned from 2004 to 2020, inviting more confounders and temporal bias as treatment strategies and approaches by individual clinicians likely changed over time. As described above, adjuvant therapy did not become standard of care until after the BILCAP trial in 2019 and the role of neoadjuvant therapy is still being studied. Analysis of the temporal distribution of chemotherapy sequence shown in Fig. 10 demonstrated that neoadjuvant therapy was uncommon prior to 2011 and the proportion of patients receiving no chemotherapy decreased over time. These results may be a reflection of patients living longer due to advances in general medical care as well as advances in chemotherapy administration. However, this context is important for interpretation of the findings of this study. Fifth, this study did not examine the role of radiation or immunotherapy, both of which may play an integral role in the future treatment of this disease.

While this study is unable to establish causality and observed survival differences should be interpreted with caution given the retrospective design and potential for confounding, these findings are hypothesis-generating and underscore the need for future prospective trials. Despite these limitations, this study provides real-world data to guide clinical decision making for this rare disease and reinforces quality standards.

## Conclusion

Gallbladder adenocarcinoma is a challenging disease for the clinician and deadly for the patient. In selected patients undergoing surgery for clinically stage IIIB gallbladder cancer, an association between reception of chemotherapy and decreased hazard of death was observed. Neoadjuvant or perioperative chemotherapy may represent an advantage over adjuvant only approaches, both in assessing tumor biology and in overall survival, though these associations did not reach statistical significance in this study. Regional lymphadenectomy was performed frequently and should remain the surgical standard in this disease process.

## Figures and Tables

**Table 1:** Patient Characteristics by Chemotherapy Sequence Class

Characteristic	Adjuvant, N = 260^1^	Neoadjuvant, N = 28^1^	No_Chemo, N = 121^1^	p-value^2^
Age	65 (11)	60(8)	72 (14)	< 0.001
Sex				0.4
Male	73 (28%)	11 (39%)	32 (26%)	
Female	187 (72%)	187 (72%)	89 (74%)	
Charlson-Deyo	186 (72%)	20 (71%)	82 (68%)	0.8
0	55 (21%)	20 (71%)	82 (68%)	
1	55 (21%)	7 (25%)	29 (24%)	
2	9(3.5%)	0(0%)	7(5.8%)	
3	10 (3.8%)	1 (3.6%)	3 (2.5%)	
Tumor Size	47(33)	39(20)	51(35)	0.4
Regional Lymphadenectomy	227 (87%)	22 (79%)	102 (84%)	0.3
Regional Nodes Examined	4.0 (4.8)	4.3 (5.6)	3.2 (7.2)	0.006
Regional Nodes Positive	1.74(1.49)	1.36(1.16)	1.64(1.87)	0.10
Margins				0.5
R0	198 (76%)	24 (86%)	98 (81%)	
R1	57 (22%)	3 (11%)	21 (17%)	
R2	5 (1.9%)	1 (3.6%)	2 (1.7%)	

1 Mean (SD); n (%)

Kruskal-Wallis rank sum test; Pearson’s Chi-squared test; Fisher’s exact test

**Table 2:** Multivariate Cox Survival Model

Characteristic	HR7	95% Cl’	p-value
Class			< 0.001
Adjuvant	—	—	
Neoadjuvant	0.76	0.46, 1.26	
No_Chemo	1.87	1.45, 2.42	
Age	1.01	1.00, 1.02	0.007
Sex			0.29
Male	–	–	
Female	0.87	0.68, 1.12	
Charlson-Deyo	1.04	0.88, 1.22	0.66
Tumor Size	1.01	1.01, 1.01	< 0.001
Regional Lymphadenectomy			0.10
No	–	–	
Yes	0.75	0.53, 1.05	
Regional Nodes Examined	0.99	0.97, 1.02	0.64
Regional Nodes Positive	1.13	1.05, 1.21	0.002
Margins			< 0.001
*R0*	–	–	
*Rl*	2.07	1.54, 2.77	
*R2*	4.05	1.91, 8.57	

^7^HR = Hazard Ratio, CI = Confidence Interval

**Table 3:** IPW-Weighted Multivariate Cox Survival Model

Variable	HR	95% CI	*p*-value
Chemotherapy Sequence			
*No Chemotherapy*	—	—	
*Adjuvant*	0.44	0.33, 0.58	< 0.001
*Neoadjuvant*	0.38	0.23, 0.62	< 0.001
Age	1.01	1.00, 1.02	0.11
Sex			
*Male*	—	—	
*Female*	1.31	0.90, 1.91	0.16
Charlson-Deyo Score	0.94	0.73, 1.21	0.63
Tumor Size (mm)	1.00	1.00, 1.01	0.19
Regional Lymphadenectomy			
*No*	—	—	
*Yes*	0.61	0.41, 0.92	0.020
Regional Nodes Examined	1.02	1.00, 1.05	0.084
Regional Nodes Positive	1.17	1.11, 1.23	< 0.001
Surgical Margins			
*R0*	—	—	
*R1*	2.98	2.06, 4.30	< 0.001
*R2*	3.91	2.35, 6.49	< 0.001

CI = Confidence Interval, HR = Hazard Ratio

## Data Availability

The data that support the findings of this study are derived from the National Cancer Database (NCDB). The NCDB is a joint project of the Commission on Cancer of the American College of Surgeons and the American Cancer Society. Access to the NCDB is restricted, and the data are not publicly available. Researchers may request access to NCDB data through the NCDB Participant User File (PUF) application process. Per NCDB data use agreements, the authors are not permitted to share the dataset used in this study.

## Abbreviations

NCDB: National Cancer Database
OS: Overall Survival
NCCN: National Comprehensive Cancer Network
ACS: American College of Surgeons
IPW: Inverse Probability Weighting
CBPS: Covariate Balancing Propensity Score
HR: hazard ratio
